# Occupations Suitable for Females

**Published:** 1880-10

**Authors:** 


### EDITORIAL DEPARTMENT.

"If thou hast truth to utter, speak;
And leave the rest to God."

OCCUPATIONS SUITABLE FOR FEMALES—There is no good and sufficient reason for the ostracism and exclusion of females from several occupations and callings which are filled almost exclusively by men. In fact, we think that all honorable trades, and occupations, in which great physical strength is not required for their proper performance, should be open to the sexes alike, and on equal terms; and for which compensation should be regulated by the efficiency and faithfulness of the work performed rather than preference for a sex, e. g. a difference is made in the amount of compensation awarded the principals of the male and female grammar schools in this City, for similar, if not identically the same kind and amount of services in each, and to perform which properly equal amount of scholarship, physical endurance and energy are required ; and yet, the female receives from a fourth, to a third less salary per annum ! Probably and equally *unjust* discrimination is made between the sexes in the teachers of lower or primary schools ! This is clearly all wrong, and an unfair discrimination against the physically weaker sex.

Few will deny the fact, that females when properly trained are the superior of males in educating children of their own sex, and it is far from being settled that they are not, *cæteris paribus* superior to male teachers in advancing small boys also, in the rudiments of education, at least. If this be so, then it follows as a matter of simple justice that *their* compensation should at least be equal to that of male teachers, for similar services! But we have only spoken of her when she performs *the same* labor as men, and if she is accounted worthy of training the manners of the young in equal degree with the other sex, why should she not be found more frequently employed in various other occupations, for which great physical strength is not demanded? Such for example as watch making, manufacturing of jewelry, telegraphing, in signal offices, type setting, etc., etc. We are not of those who favor her entrance into the professions, not however because of any lack of mental power to grasp and comprehend the more obstruce and intricate problems that require to be solved, and appreciated by those who would master them, and attain to eminence in them. But, because of the high, perhaps slightly ideal ground, we would have her occupy as the mistress of our heart's purest and dearest affections, the crystal fountain to which in our hour of weakness, temptation or despair, we can turn, though polluted and clothed with sinful deeds, for her elevating and purifying influence on our conscience and our hearts ! We would have her always hold the endearing places of mother, wife or sister in our affections, and keep her the presiding diety in our homes, as well as in our hearts ; pure, gentle, confiding, loving being that she always is, unless forced by circumstances into the arena of the active world, and to feel that man alone is vile. We know of some pure and " womanly " women in our profession, through whose intelligence and industry it is beautified and adorned. And we are prepared to extend a hearty greeting at all times to such worthy female members. But still we do not wish those whom we delight to honor and to love so well, to be doctors.

